# Amphiphobic Nanostructured Coatings for Industrial Applications

**DOI:** 10.3390/ma12050787

**Published:** 2019-03-07

**Authors:** Federico Veronesi, Giulio Boveri, Mariarosa Raimondo

**Affiliations:** Institute of Science and Technology for Ceramics ISTEC CNR, via Granarolo, 64-48018 Faenza, Italy; federico.veronesi@istec.cnr.it (F.V.); giulio.boveri@istec.cnr.it (G.B.)

**Keywords:** amphiphobicity, hybrid coatings, anti-soiling, snow-repellent, friction reduction

## Abstract

The search for surfaces with non-wetting behavior towards water and low-surface tension liquids affects a wide range of industries. Surface wetting is regulated by morphological and chemical features interacting with liquid phases under different ambient conditions. Most of the approaches to the fabrication of liquid-repellent surfaces are inspired by living organisms and require the fabrication of hierarchically organized structures, coupled with low surface energy chemical composition. This paper deals with the design of amphiphobic metals (AM) and alloys by deposition of nano-oxides suspensions in alcoholic or aqueous media, coupled with perfluorinated compounds and optional infused lubricant liquids resulting in, respectively, solid–liquid–air and solid–liquid–liquid working interfaces. Nanostructured organic/inorganic hybrid coatings with contact angles against water above 170°, contact angle with n-hexadecane (surface tension γ = 27 mN/m at 20 °C) in the 140–150° range and contact angle hysteresis lower than 5° have been produced. A full characterization of surface chemistry has been undertaken by X-ray photoelectron spectroscopy (XPS) analyses, while field-emission scanning electron microscope (FE-SEM) observations allowed the estimation of coatings thicknesses (300–400 nm) and their morphological features. The durability of fabricated amphiphobic surfaces was also assessed with a wide range of tests that showed their remarkable resistance to chemically aggressive environments, mechanical stresses and ultraviolet (UV) radiation. Moreover, this work analyzes the behavior of amphiphobic surfaces in terms of anti-soiling, snow-repellent and friction-reduction properties—all originated from their non-wetting behavior. The achieved results make AM materials viable solutions to be applied in different sectors answering several and pressing technical needs.

## 1. Introduction

Nowadays, repellence to liquids and/or fluids is considered a key aspect of materials engineering. The coexistence on the same material of superhydrophobicity together with oleophobicity, termed amphiphobicity, significantly improves its ability to answer relevant industrial needs, for which the repellence against liquids and/or fluids in a wide range of surface tension could introduce innovation at different levels (single components, devices, whole equipment, etc.). 

The analysis of the current state of the art suggests that surfaces with a reduced wettability can be generated by many routes combining different approaches and strategies [1,2,3,4,5]. Depending on the materials typology, they include electrospinning [6], layer-by-layer (LbL) deposition [4], anodization [7], electrochemical approaches [8], and chemical vapor deposition (CVD) [9]. However, hindrances to their practical application still exist, mainly concerning the plethora of processing steps and the affordability of procedures and equipment. In addition, the realization of durable non-wetting surfaces, suitable for different environmental conditions, is still a great challenge to be overcome. In some cases, in fact, even moderate forces or specific chemical environments could destroy the surface layers, even leading to complete loss of their performance [10,11,12,13]. To overcome some of the issues of superhydrophobic surfaces, new strategies have been proposed recently such as the modification of the working interface, from solid to liquid, by realizing so-called Slippery Liquid-Infused Porous Surfaces (SLIPSs) [14,15,16]. 

This work deals with the synthesis and application on metals and alloys of hybrid layers produced by a first deposition of nanoscale-featured inorganic components, obtained via sol–gel, followed by the subsequent “hybridization” with an organic moiety to tailor the surface chemical composition. In particular, functional surfaces were obtained following both the so-called Lotus leaf (LF) approach [17] and the SLIPS route, depositing a Al_2_O_3_ nanoparticles suspension via dip coating followed by chemical modification with a fluoroalkylsilane solution (FAS) in order to reduce surface energy (SE). SLIPSs were obtained by the further covering of the chemically modified Al_2_O_3_ nanostructured layer with a fluorinated lubricant (Krytox® GPL 100), creating a new liquid surface. All the materials have been deeply characterized in terms of wetting behavior (water and oils contact angles, contact angle hysteresis), surface energy, chemistry (X-ray photoelectron spectroscopy (XPS) analyses) and microstructure by field-emission scanning electron microscope (FE-SEM) observations. Furthermore, we assessed the snow-repellent and anti-soiling behavior of coated surfaces by means of outdoor exposure in extreme environments, as well as their anti-friction properties by tests in an ad hoc designed rig. Results suggest that the nanostructured hybrid coating deposited on metals and alloys present a greatly reduced wetting against water and oils/lubricants that, together with the good durability, make them suitable candidates for industrial applications.

## 2. Materials and Methods

### 2.1. Preparation of Alumina Sols

Alcoholic alumina sol was prepared following the literature [18]. Aluminum-tri-sec-butoxide (97%, Sigma-Aldrich, St. Louis, MO, USA) was stirred in isopropyl alcohol (99%, Sigma-Aldrich) for 1 h at room temperature. Then, ethyl acetoacetate (99%, Sigma-Aldrich) as the chelating agent was added and the solution was stirred for 3 h. Finally, water was gradually added to the solution to promote the hydrolysis of the alkoxide, and then the sol was stirred for 24 h at room temperature. The molar ratios of isopropyl alcohol, chelating agent and water with respect to Al were set to 20, 1 and 4, respectively. The particle size distribution of the suspension was evaluated by a dynamic light scattering (DLS) analyzer (Zetasizer Nano S, Malvern Instruments, Malvern, UK) working in backscattering mode (2θ = 173°) at 25 °C. 

**Figure 1:**
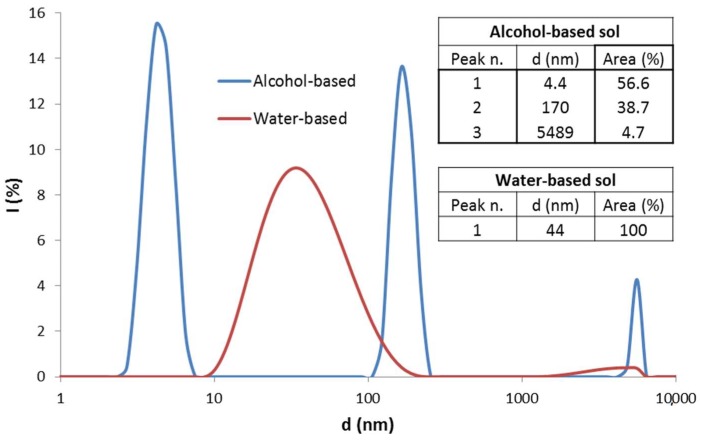
Size distribution of alumina nanoparticles in the alcohol-based (blue line) and in the water-based (red line) sol. Inset tables report the average size for each peak and the related area percentage.

The alcohol-based sol showed a multimodal particle size distribution (Figure 1, blue curve). The peak with the highest scattering intensity is centred at 4 nm with smaller amounts of aggregated particles.

An aqueous suspension of alumina nanoparticles was also synthesized [19]. Ethyl acetoacetate was added into deionized water and the mixture was stirred and heated until the temperature reached 70 °C. Then, aluminum-tri-sec-butoxide was added. After complete solubilisation, nitric acid (0.5 M) was added dropwise to catalyse peptization and the mixture was kept under stirring at 70 °C for 24 h. The molar ratios of water, chelating agent and nitric acid with respect to Al were set to 90:1, 1:1 and 0.3:1, respectively. The pH of the as-prepared sol was 3.5. DLS particle size distribution showed a single peak with an average particle size of 44 nm (Figure 1, red line).

### 2.2. Deposition of Hybrid Coatings on Different Substrates

According to different applications to be envisaged, aluminum foils (Al 1050, 100 × 50 × 2 mm^3^) and cables (Anticorodal^®^ wires with a diameter of about 3 cm) as well as brass slippers (CuZn_40_Al_2_ alloy) were used as substrates. In order to maximize coating adhesion [20,21], samples were sandblasted with glass microspheres (Swarco, 400–800 µm diameter) to reach roughness R_a_ of about 3–4 µm. Typically, they were initially cleaned with soapy water followed by ethanol to remove impurities, then dip coated in the alcohol- or water-based alumina sols with a withdrawal speed of 2 mm/s and soaking time of 5 s. After drying at room temperature, they were annealed at 300 °C for 1 h to get an amorphous, dense layer of Al_2_O_3_, then immersed in boiling water for 30 min to form flaky boehmite and thermally treated again (10 min) to stabilize the inorganic film. Subsequently a commercial fluoroalkysilane solution (Dynasylan® SIVO CLEAR EC, Evonik, Essen, Germany) was dip-coated on the boehmite layer (dipping–withdrawing speed: 2 mm/s, soaking time: 120 s), followed by consolidation at 150 °C for 30 min, obtaining Lotus leaf-like (LF) surfaces. In order to produce SLIPS samples, LFs were immersed in a lubricant oil (Krytox GPL 100, DuPont, Wilmington, DE, USA) for 5 min, followed by drying at room temperature.

### 2.3. Surface Characterization 

The static water contact angle (WCA) and the static contact angle with n-hexadecane (CA_n-hexa_) of aluminum samples were detected with sessile drops (volume 6 μL) using an optical contact angle system (Drop Shape Analyzer DSA30, Krüss GmbH, Hamburg, Germany). The dynamic wetting behavior, expressed by the CA hysteresis (CAH), was calculated as the difference between the advancing and the receding contact angle of a sessile drop (starting volume of 10 μL). CAH is related to adhesion [22]: the higher the CAH, the stronger the adhesion between liquid drops and solid surface. Contact angle values labelled in Table 1 have been calculated as the average of five to ten measurements on different points of the surface. Surface energy (SE) of the coated surfaces was calculated with the OWRK method [23] using water and n-hexadecane as reference liquids. The value of SE total energy results as the sum of polar (due to hydrogen bonding and dipole–dipole interactions) and dispersive components. 

The wetting behavior of coated brass slippers was evaluated in terms of static contact against Arnica-46 lubricant oil (Agip, Rome, Italy) and compared with that of the uncoated specimen. Arnica-46 oil has a surface tension γ of 29.4 mN/m and represents one of the most widely used lubricants for hydraulic systems.

The surface morphology was investigated with a FE-SEM (Gemini Columns SIGMA Zeiss, Oberkochen, Germany). Surface chemical composition of coated surfaces was investigated with XPS. Measurements were performed with a modified Omicron NanoTechnology MXPS system equipped with a monochromatic X-ray source (Omicron XM-1000, Houston, TX, USA), a dual X-ray anode (Omicron DAR 400), and an Omicron EA-127−7 energy analyzer. Al Kα photons (hν = 1486.7 eV) or Mg Kα photons (hν = 1253.6 eV), both generated operating the anode at 14−15 kV and 10−20 mA, were used for excitation. Further experimental details can be found elsewhere [24].

### 2.4. Durability Tests

Abrasion resistance of the coated samples was checked according to UNI EN 1096-2 standard [25]. This method requires to apply loads of 4 N for a short time (30 s) by an abrasive felt disk (diameter of 60 mm) rotating on the test sample at a speed of 60 rpm.

Wet chemical tests in different harsh environments were carried out by immersion of the functionalized samples in basic (sodium hydroxide, pH = 13), acid (acetic acid, pH = 3), saline (sodium chloride 100 g/L, pH = 7.0) and seawater-mimicking (pH = 7.9, alkalinity degree equal to 230 g/m^3^, sulfates amount of 2225 g/m^3^ and sodium chloride amount of 35 g/L) solutions. After 60 days, they were withdrawn, rinsed and characterized to assess changes in WCA, CAH and SE values. The resistance to ultraviolet (UV) radiation was checked keeping the samples under an OSRAM Ultra-Vitalux 300W lamp at an irradiation intensity of 5.0 ± 0.2 mW/cm^2^ for 30 min. The wetting performances were then checked after cooling the samples for 20 min at room conditions.

### 2.5. Assessment of Snow-Repellent and Anti-Soiling Behavior in Outdoor Environments

Aluminum cylindrical cables with a diameter of about 3 cm, commercially known as Anticorodal^®^ wires, were coated either as received (R_a_ < 0.3 m) or after sandblasting (R_a_ up to 3–4 m) following both LF and SLIPS design routes. Samples with different surface roughness were tested as R_a_ is known to be a relevant parameter in icing phenomena [26,27]. Subsequently they have been exposed during the last winter season at an outdoor test facility (OTF) located in the Western Italian Alps, at an altitude of 950 m a.s.l. For comparison, cables bearing different surface treatments—smooth and sandblasted uncoated cables, as well as commercial, silicone-based Nusil™1009 and Nusil™1082 treatments—were exposed [28]. During the snowfall events, different atmospheric conditions were recorded in terms of outdoor temperature (−2 °C < T < 0 °C) and liquid water content. 

Anti-soiling ability of aluminum foils, located on a roof in the city of Karachi (Pakistan), was assessed by direct exposure for two months, under the local hot climate, to dust and polluted air. In addition to visual examination, the wetting performances and SE of coated samples, before and after exposure, were compared with those of untreated materials which underwent the same conditions. 

### 2.6. Anti-Friction Tests

**Figure 2:**
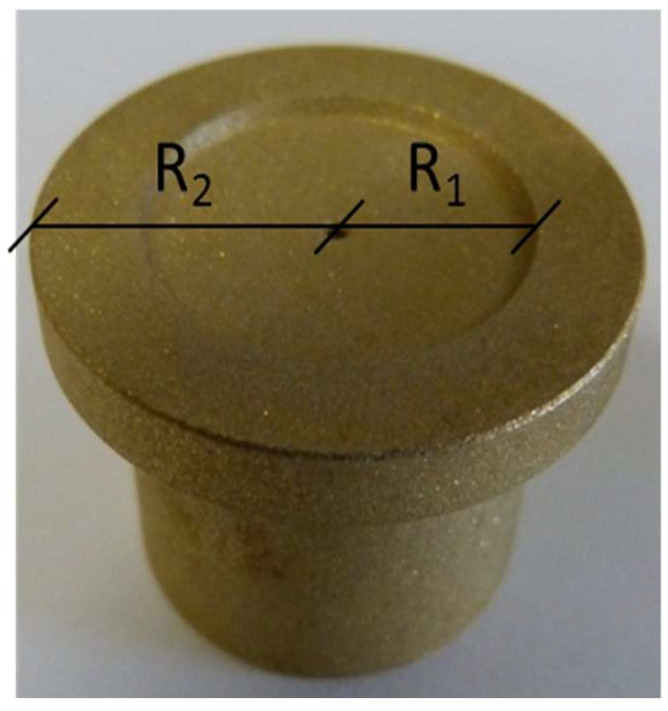
Sandblasted brass slippers. Inner radius *R*_1_ and outer radius *R*_2_ are reported.

**Figure 3:**
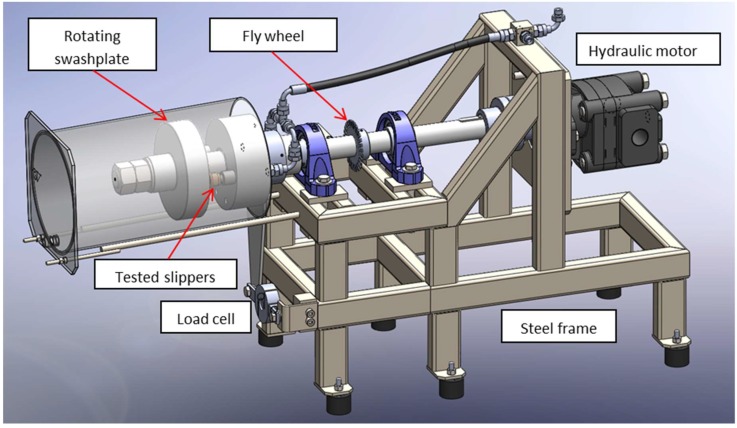
Test rig for the assessment of friction on brass slippers in hydrodynamic lubrication regime.

Anti-friction properties of coated, sandblasted brass slippers (CuZn_40_Al_2_ alloy, inner radius *R*_1_ = 9.25 mm, outer radius *R*_2_ = 14.15 mm (Figure 2) were tested in the tailored test rig sketched in Figure 3 [29], designed and realized at CNR-IMAMOTER (Ferrara, Italy). The rig is able to simulate the real operational conditions of an axial piston pump in its starting stages. 

Typically a pair of slippers was inserted in the test rig and, during the tests, lubricant oil was pumped through the slippers holes with pressure *p*_0_ leaking onto the slipper surface. Then, the swashplate was approached to the slippers while rotating at a certain rotational speed *v*, thus establishing a hydrostatic lubrication regime. A load cell was used to measure the friction force *F_f_* acting on the slippers and the friction coefficient *C_f_* was calculated as the ratio between *F_f_* and the force applied from the slippers to the swashplate *F*:

$$F=p_{0}\frac{\pi }{2}\frac{R_{2}^{2}-R_{1}^{2}}{log\frac{R_{2}}{R_{1}}},\;C_{f}=\frac{F_{f}}{F}$$

Three types of functional tests were performed:

- Constant *v* = 1000 rpm and variable *p*_0_ = 10 ÷ 100 bar;
- Constant *p*_0_ = 50 bar and variable *v* = 300 ÷ 1800 rpm;
- Endurance tests for 1200 min at constant *p*_0_ = 50 bar and *v* = 1000 rpm.

## 3. Result and Discussion

### 3.1. Wettability Performances of Coated Samples

Compared to untreated surfaces, aluminum samples coated according to LF and SLIPS approaches exhibited an increased repellence against both water and n-hexadecane, coupled with an enhanced dynamical water repellence (low values of CAH). The contact angle values for all samples are shown in Table 1.

In addition, the data clearly indicate a significantly different behavior between LF and SLIPS samples. The first ones exhibited higher static repellence against water (WCA up to 172°) and n-hexadecane (CA_n-hex_ higher than 140°) with respect to the SLIPS ones (whose WCA and CA_n-hex_ values are, respectively, 121° and 100°). With regard to CAH values, SLIPS presented the most outstanding dynamic behavior against water with CAH_w_ much lower than 5° and no residual sign of drop sticking. The dynamic repellence against n-hexadecane is also different according to the design approach to superhydrophobicity/oleophobicity. While LF surfaces are not effective in promoting the mobility of n-hexadecane drop, SLIPS behave much better with CAH_n-hex_ of about 10°. 

The SE of all coated surfaces was lowered, this involving a remarkable repellence against liquids with surface tension values down to 27 mN/m such as n-hexadecane. These effects are due to the efficient coupling between the surface nano-structuring and the hybridization subsequent to the deposition of fluorine-bearing moieties. We also verified that samples produced by the only deposition of organic layer, thus lacking of the proper nanostructure provided by boehmite, exhibited WCA not higher than 130° and strong adhesion of water drops, with CAH_w_ higher than 60°. This circumstance confirms the key role of the ceramic oxide nanostructure in the enhancement of CA values towards the upper limits of superhydro- and/or oleophobic behavior.

### 3.2. Surface Morphology of Coated Samples

**Figure 4:**
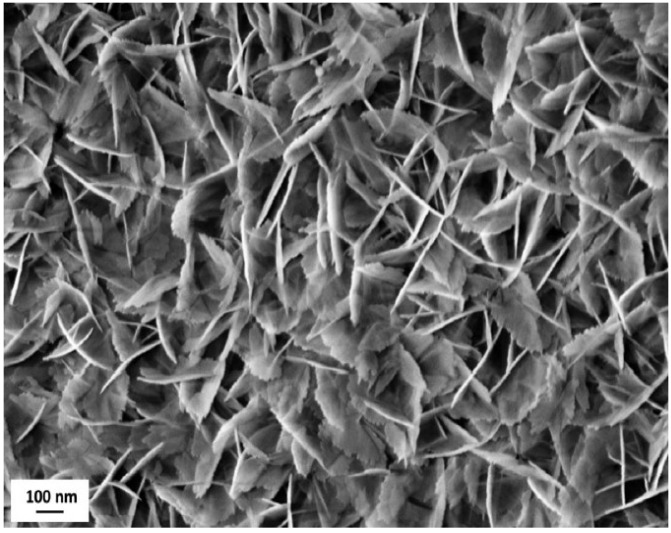
Field-emission scanning electron microscope (FE-SEM) image of flower like morphology obtained after coating the samples’ surface with the Al_2_O_3_ nanoparticles sol, followed by the boiling treatment. Scale bar is reported.

The inorganic layer morphology consists of nano-sized boehmite lamellae, randomly assembled in a typical flower-like structure, as highlighted by FE-SEM analysis (Figure 4). 

The boehmite flakes in Figure 4 have a length of about 200 nm. The random aggregation of flakes creates small voids whose size (30–60 nm) is consistent with the dimensions generally considered as recommendable to provide functional layers with the Cassie–Baxter wetting state [30]. Notably, the addition of fluoroalkylsilane and Krytox lubricant layers did not significantly affect surface morphology.

**Figure 5:**
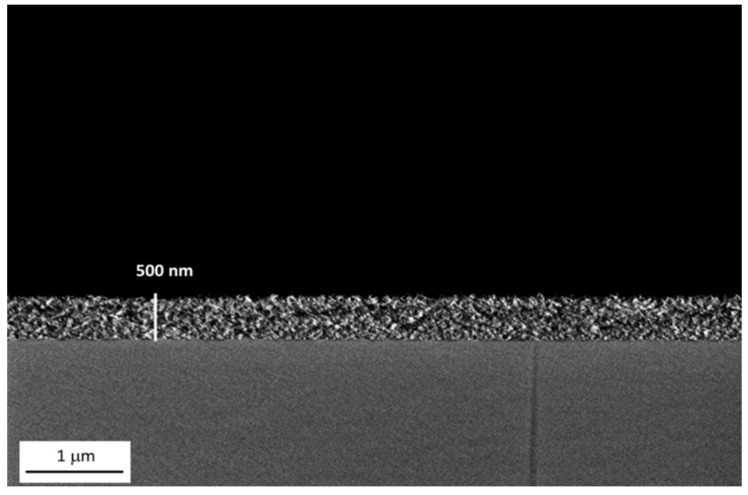
FE-SEM image of the boehmite film thickness. Scale bars are reported.

The average thickness of alumina coating deposited via dip coating (withdrawal speed 2 mm/s and soaking time 5 s) is around 500 nm as highlighted in Figure 5. Again, the further deposition of the organic (LF samples) or lubricant (SLIPS samples) outer layer did not lead to a relevant change in coating thickness. 

### 3.3. Surface Chemical Composition

**Figure 6:**
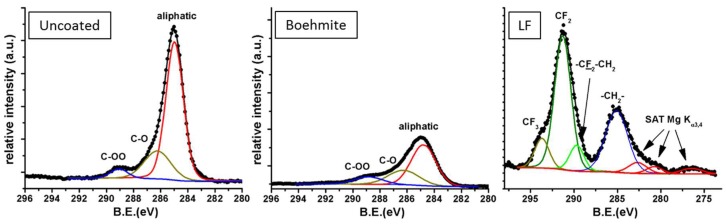
C1s portions of the X-ray photoelectron spectroscopy (XPS) spectra for uncoated (**left**), boehmite-coated (**middle**) and LF-coated aluminum (**right**).

XPS measurements were performed on boehmite-, boehmite plus fluoroalkylsilane-coated surfaces (LF approach) as well as on uncoated samples taken as reference. Focusing on the C1s portion of the spectrum (Figure 6), it is possible to acknowledge the evolution of carbonaceous moieties on the surface. While some aliphatic and carbonyl/carboxylic pollutants were detected on uncoated aluminum (left-hand spectrum), alumina deposition and treatment in boiling water allowed these pollutants to be mostly removed (middle spectrum). Finally, grafting fluorinated groups like –CF_3_ and –CF_2_– were provided by fluoroalkylsilane (right-hand spectrum). Remarkably, the commercial fluoroalkylsilane solution contained non-perfluorinated molecules, as hinted at by the presence of –CH_2_– group peaks in the spectrum. Even more detailed analysis of the XPS spectra can be found elsewhere [24].

### 3.4. Durability Tests

**Figure 7:**
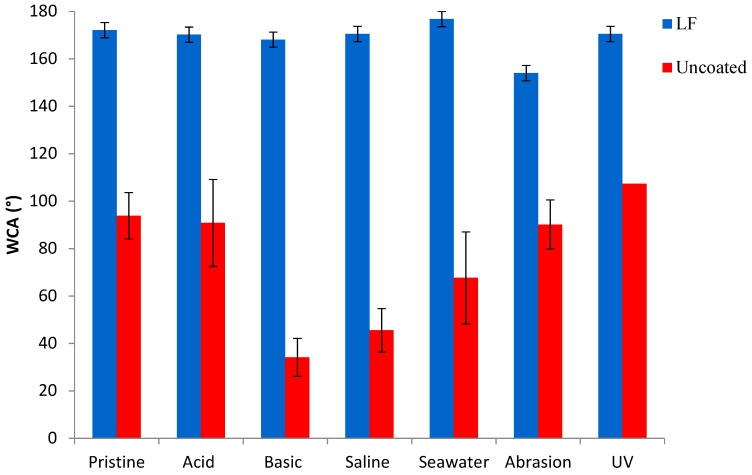
Water contact angle (WCA) of uncoated (in red) and LF (in blue) samples before (“pristine”) and after ageing tests.

**Figure 8:**
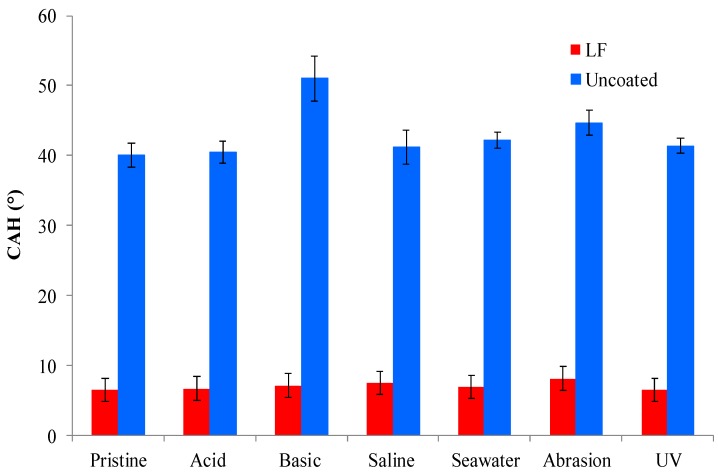
Contact angle hysteresis (WCA) of uncoated (in red) and LF (in blue) samples before (“pristine”) and after ageing tests.

**Figure 9:**
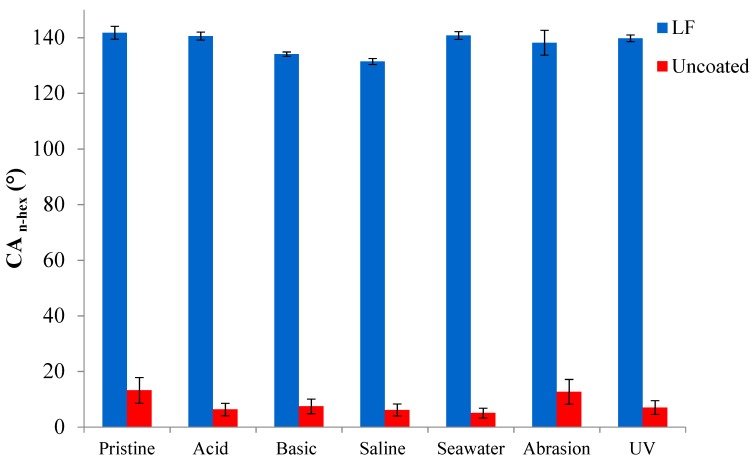
Contact angle against n-hexadecane (CA_n-hex_) of uncoated (in red) and LF (in blue) samples before (“pristine”) and after ageing tests.

Figure 7, Figure 8 and Figure 9 show, respectively, the trends of WCA, CAH and CA_n-hex_ for both uncoated and LF surfaces before (sample labelled as pristine) and after the simulation of different working and wearing conditions. In the present section, the discussion is only referred to LF surfaces as, notwithstanding the testing of SLIPS is still ongoing, the trends of LF and SLIPS are similar. 

The following are the main evidence, to be extended to SLIPS samples too:

1. The hybrid coating clearly acts as a protector layer for the metal substrate. LF samples provide a survey of chemical and mechanical stability as they retain their WCA values after the tests. In the same time span, uncoated aluminum surfaces displayed a remarkable decrease in WCA when immersed in basic, saline and seawater-mimicking solutions. These effects are due to the reactivity of aluminum in such chemically aggressive environments [31].
2. Notwithstanding that some of the adopted ageing conditions are known to be particularly deleterious to preserve static performances and, above all, de-wetting ability [19], coated samples kept their CAH substantially unchanged after immersion in acidic and seawater solutions, as well under UV irradiation. CAH values suffered a very limited increase only after soaking in basic and saline solutions and after abrasion test. In this latter case, the decreasing of WCA is limited to about 15%.
3. Oleophobic behavior of coated samples, expressed by their CA_n-hex_, is substantially unaffected by the different chemical environments, as well as by wearing and UV exposure, while CA_n-hex_ of uncoated ones decreases largely, except for abrasion tests. This represent a relevant issue in order to forecast the potential industrial applications of coated surfaces.

**Figure 10:**
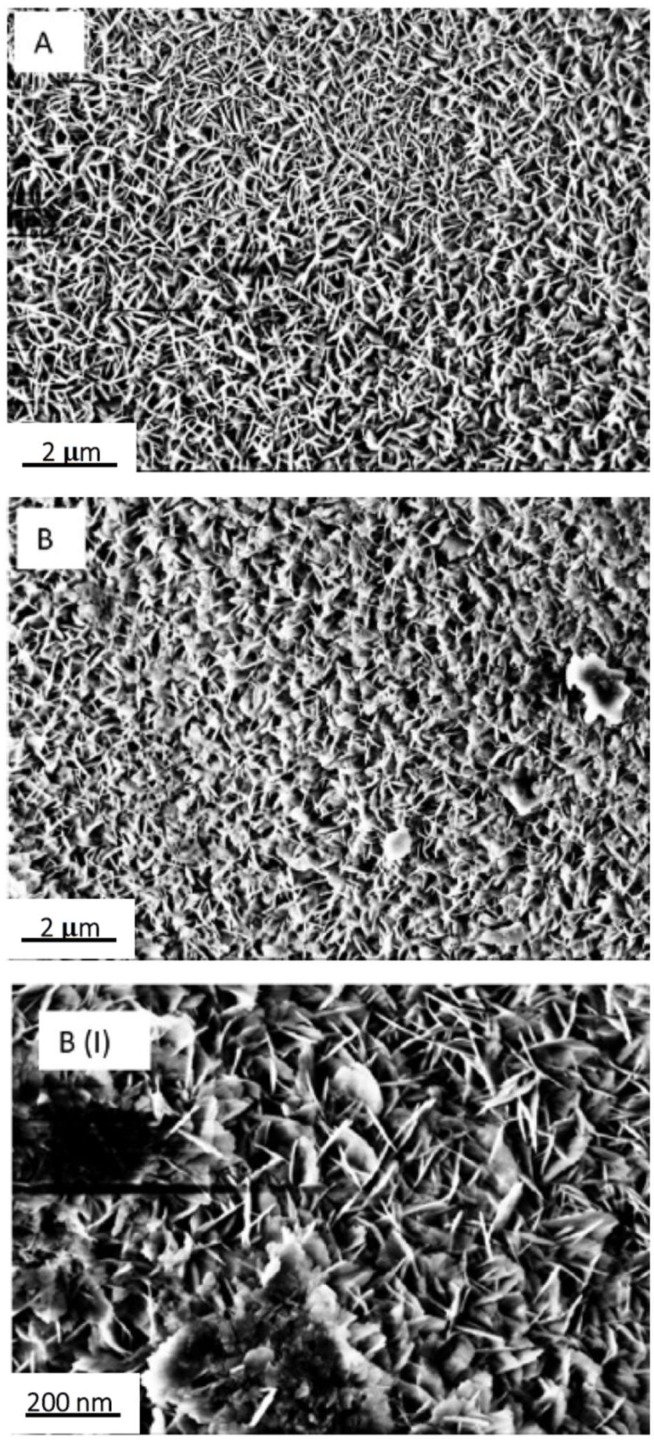
FE-SEM images of S sample (**A**) before and (**B**) after abrasion test. The greater magnification of B (labelled **B(I)**) highlights the presence of some damage.

The FE-SEM images of coated (LF) surfaces before and after abrasion (Figure 10) confirm that the flower-like structure was maintained after the test, even if a certain number of defects have been introduced. This partial removal of the nano-structure can explain the little decrease of wetting performance after abrasion process, coupled with an increase in standard deviation, meaning the introduction of a greater number of defects in the coating.

### 3.5. Snow-Repellent Behavior

**Figure 11:**
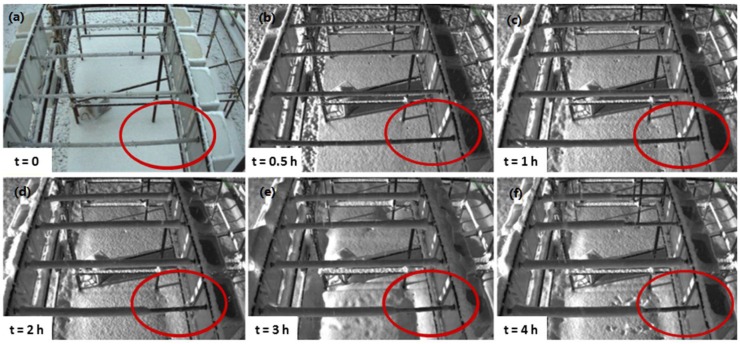
Pictures of coated cables exposed under snowfall condition in outdoor test facility. The red circle highlighted the sandblasted, LF-coated cable with no snow accumulation. Starting time (t = 0) was established when the snowfall began. (**a**) t = 0; (**b**) t = 0.5 h; (**c**) t = 1 h; (**d**) t = 2 h; (**e**) t = 3 h; (**f**) t = 4 h.

The evolution of snow accumulation on anticorodal cables with different surface treatments is shown in Figure 11. Twelve samples were exposed on four rows of three samples each:

- First row (top): (from left to right) smooth uncoated, smooth Nusil™ 1082, smooth Nusil™ 1082;
- Second row: smooth uncoated, smooth Nusil™ 1009, smooth Nusil™ 1009;
- Third row: smooth uncoated, smooth SLIPS, smooth LF;
- Fourth row: sandblasted uncoated, sandblasted SLIPS, sandblasted LF.

During the recorded snowfall, which occurred at a temperature of about −2 °C (thus involving dry snow with a low liquid content, LWC, and almost spherical snowflakes), sandblasted LF cable (red circles in Figure 11) showed a significant delay in snow deposition and subsequent snow layers accretion. This behavior is even more relevant when compared to that of the other coated cables under investigation, whether smooth or sandblasted. Remarkably, smooth, LF-coated cables (third line of samples, right side) did not display the same delay in snow accumulation as their sandblasted homologues. However, further observations revealed that in different conditions (e.g., wet snow occurring at higher temperature and with higher LWC), this behavior was no longer observed. Thus, a better understanding of the relationships between snow layer accretion and materials design parameters needs to be achieved. The loss of the snow-repellent performances can be probably ascribed to the progressive damaging of the coating caused by the exposure in the harsh winter environment. 

In this case, SLIPS surfaces behave in a different way with respect to the LF ones, showing a much lower ability to avoid snow accumulation. The lower snow-repellent performances of SLIPS surfaces correspond to a different drop rebound behavior. Different studies in literature confirm that the contact time between the snowflakes and the surface plays a key role on ice and snow accumulation [32,33] being directly linked to the permanence of snow/ice particles on the surfaces. Previous studies [34] showed that LF samples present a complete rebound of the water drops with very short contact times, i.e., in the order of milliseconds. We might infer that snowflakes do not have enough time to deposit on LF surfaces, thus leading to snow detachment. On the other hand, on SLIPSs snowflakes require much longer times for removal, eventually leading to snow accumulation.

### 3.6. Anti-Soiling Ability

**Figure 12:**
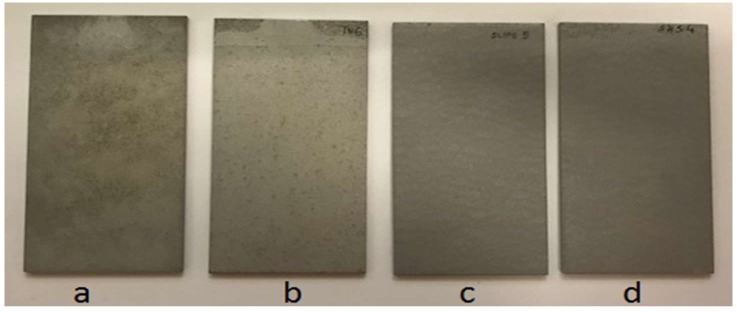
Dust deposition on uncoated (**a**,**b**) and coated (**c**), SLIPS; (**d**) LF aluminum substrates.

The deposition of amphiphobic coatings on aluminum substrates leads to a reduction of soil deposition with respect to uncoated samples (Figure 12). 

**Figure 13:**
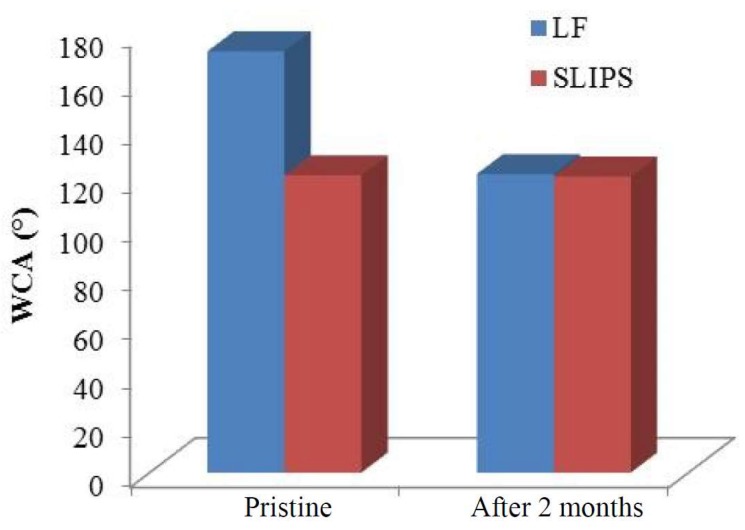
Water contact angle (WCA) of the coated aluminum samples before (in blue) and after 2 months (in red) of exposure in Karachi, Pakistan.

According to the different typologies of amphiphobic surfaces, their wettability before and after exposure in real environment (Figure 13) revealed that, notwithstanding SLIPS surfaces presented a lower water repellence (WCA of 121.4 against 172 of LF samples), they preserved this value after two months of exposure in heavily soiled environment. On the other hand, LF surfaces decreased their WCA to about 120°.

**Figure 14:**
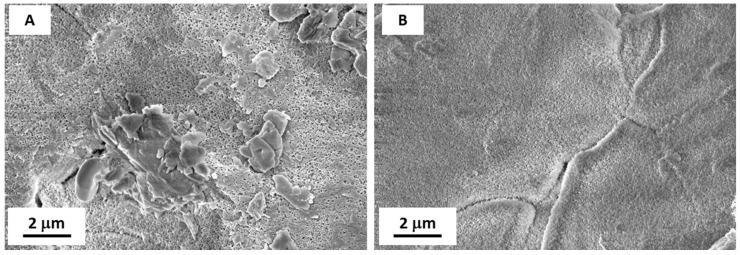
FE-SEM images of LF (**A**) and SLIPS samples (**B**) after 2 months of exposure in Karachi, Pakistan. Scale bars are reported.

FE-SEM observations of LF and SLIPS samples after exposure (Figure 14) helped us to understand such behavior. As seen in Figure 14A, LF surfaces showed a relevant accumulation of micron-sized particles which interfere with the Cassie–Baxter wetting state observed on pristine LF samples. Therefore, their WCA significantly decreased after exposure. On the other hand, SLIPSs displayed little to no sticking of dust particles on their surface (Figure 14B), maintaining their peculiar surface morphology and wetting properties unaltered.

The actual relationship between the non-wetting behavior and anti-static properties of these surfaces is still unknown, but it represents the biggest motivation of our upcoming activities. More comprehensive results are expected in the next months after further observations and the measurement of surface charge, which might significantly change due to the presence of coatings.

### 3.7. Anti-Friction Performance

The deposition of LF coating proved able to radically reverse the wetting behavior of brass slippers. In fact, uncoated slippers showed remarkable oleophilicity with respect to Arnica46 lubricant oil, with a contact angle of 17°. Contact angle values did not change after sandblasting. However, LF coating promoted a significant increase in oil repellence, with contact angle with Arnica46 reaching 124°. Thus, the coating was able to induce oleophobicity also on brass slippers.

**Figure 15:**
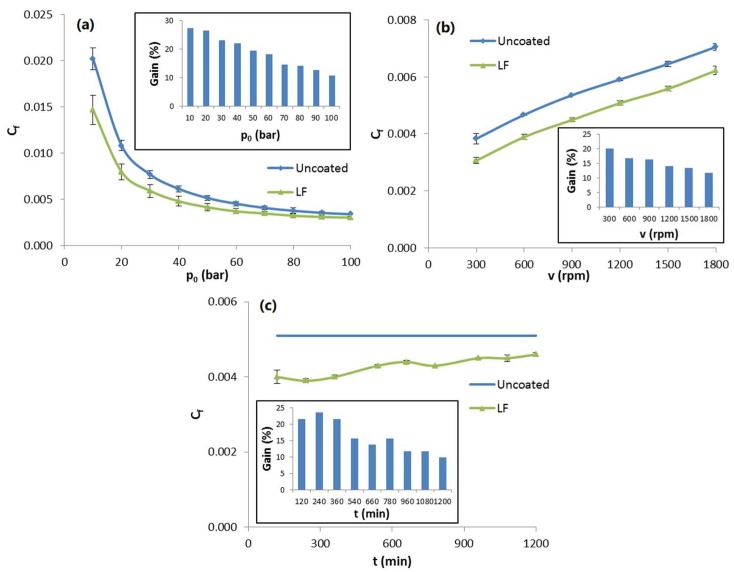
Friction coefficient *C_f_* measured during functional tests with variable pressure (**a**), variable rotational speed (**b**) and endurance tests (**c**) for LF slippers (green triangles) and uncoated ones (blue squares). Every inset reports the evolution of percentage gain in *C_f_* for LF samples compared to reference uncoated slippers.

Functional tests showed interesting results, as shown in Figure 15. LF samples (green triangles) showed significant friction coefficient reduction in all tests compared to uncoated slippers (blue squares). In variable *p*_0_ tests (top left), the gain in *C_f_* compared to uncoated slippers ranged from 27% at 10 bar down to 11% at 100 bar. A similar trend was observed in variable *v* tests (top right), with the gain ranging from almost 21% at 300 rpm to 12% at 1800 rpm. It is worth mentioning that these conditions are perfectly representative of the real operational conditions experienced by slippers during the starting phases, when pressure and speed increase. For the slippers surface, these are the most stressful phases, as friction coefficient is at its highest.

Most remarkably, endurance tests showed that the LF coating is able to withstand over 1200 min of such harsh conditions (bottom), which usually last less than a minute in real pumps. In fact, friction coefficient reduction was mostly unaltered at the end of the test, with a gain in *C_f_* close to 10%.

**Figure 16:**
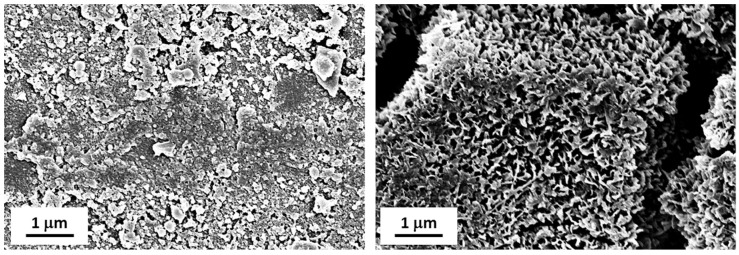
FE-SEM observations of LF slippers after endurance tests in the outer area (“crown”, **left**) and in the central area (close to the hole, **right**). Scale bars are reported.

The surface of the coated slippers was observed after endurance tests and resultant images are displayed in Figure 16. On the outer part of the slipper top surface, e.g., the so-called “crown”, the coating was visibly damaged, with clear scratches and signs of abrasion. However, in the central area close to the hole the coating was unaltered, maintaining its flower-like nanostructure. We hypothesize that the presence of such unspoiled coating is responsible for the friction reduction observed at the end of the endurance tests. Further investigations on the friction reduction properties of SLIPS are underway in the same test rig.

## 4. Conclusions

This work reveals the efficiency of different thin coating technologies (in the present work referred as LF and SLIPS) to achieve repellent aluminum and alloys substrates against water and n-hexadecane, from both static and dynamic points of view, thanks to the coupling between structural modification and the alteration of surface chemistry. Outstanding features concerning the resistance of coated alloy in different environmental conditions come out as the most important result, matching some of the basic requirements related to the utilization of functional surfaces in many industrial fields and bringing about a revolutionary strategy into the design of aircrafts, hulls, propulsion components, just to give some examples.

The assessment of snow-repellent ability revealed a better efficiency of the LF surfaces, probably due to the lower contact time between wires and snowflakes compared with the SLIPS samples. 

Anti-soiling tests in Pakistan exhibited a huge soil deposition reduction for both LF and SLIPS surfaces, but only the last ones maintained their WCA unchanged after exposure. In order to better understand the anti-soiling mechanism, surface charge analysis will be performed on anti-dust samples, before and after exposure in Karachi.

Furthermore, functional tests in a specific test rig simulating an axial piston pump showed a remarkable and lasting friction coefficient reduction when a LF coating was applied to the surface of brass slippers, switching its wetting behavior from oleophilic to oleophobic.

All these functional tests prove once again the importance and future potential of amphiphobic coatings in several application fields and the need to properly tailor their properties considering the specific operational conditions.

## Figures and Tables

**Table 1:** Static contact angle against water (WCA) and n-hexadecane (CA_n-hex_), contact angle hysteresis against water (CAH_w_) and n-hexadecane (CAH_n-hex_), surface energy (SE) of uncoated and coated aluminum samples. Standard deviation (s.d.) is reported as error.

Sample	WCA ± s.d.	CAH_w_ ± s.d.	CA_n-hex_ ± s.d.	CAH_n-hex_ ± s.d.	SE (mN/m)
Uncoated	93.8 ± 3.2	40.0 ± 3.1	13.2 ± 2.3	-	31.3
LF	172.1 ± 1.8	6.5 ± 1.7	141.8 ± 6.2	77.1 ± 6.2	0.65
SLIPS	121.4 ± 1.4	1.1 ± 0.7	100.4 ± 4.4	10.1 ± 1.7	12.7
